# Enzymes Encapsulated within Alginate Hydrogels: Bioelectrocatalysis and Electrochemiluminescence Applications

**DOI:** 10.1021/acs.analchem.2c03389

**Published:** 2022-11-08

**Authors:** Lucia Simona Ferraraccio, Donatella Di Lisa, Laura Pastorino, Paolo Bertoncello

**Affiliations:** † Department of Chemical Engineering, Faculty of Science and Engineering, Swansea University, Bay Campus, Crymlyn Burrows, Swansea SA1 8EN, U.K.; ‡ Centre for NanoHealth, Swansea University, Singleton Campus, Swansea SA2 8PP, U.K.; § Department of Informatics, Bioengineering, Robotics and System Engineering, University of Genova, Via Opera Pia 13, 16145 Genova, Italy

## Abstract

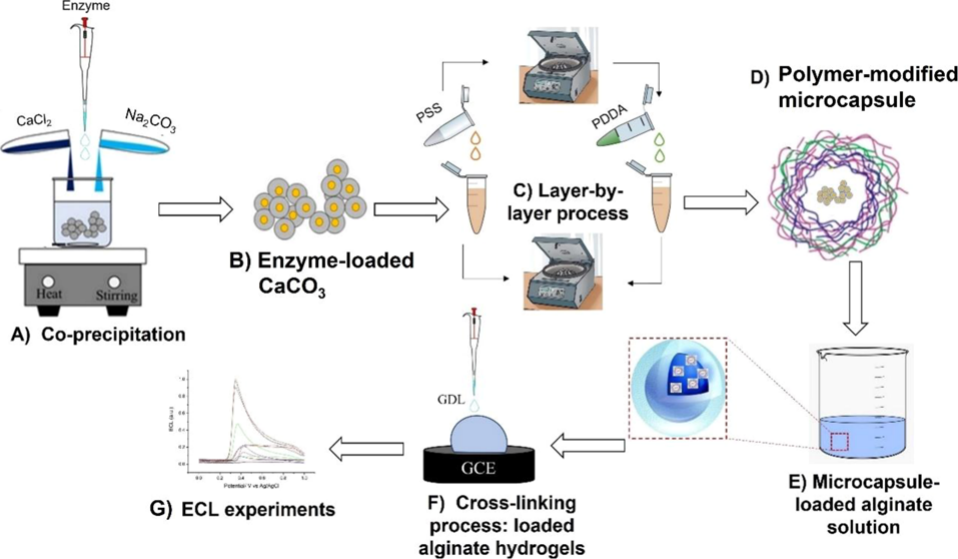

A simple procedure to incorporate enzymes (horseradish peroxidase, HRP, and lactate oxidase, LOx) within alginate hydrogels is reported with electrochemiluminescence (ECL) used to detect the enzymatic reactions with the corresponding substrates. First, HRP and LOx were successfully immobilized into CaCO_3_ microspheres, followed by the electrostatic layer-by-layer deposition of a nanoshell onto the microspheres, and finally by their dispersion into alginate solution. The as-prepared dispersion was drop cast onto the glassy carbon electrodes and cross-linked by the external and internal gelation methods using Ca^2+^ cations. The enzymes encapsulated within the alginate hydrogels were characterized using cyclic voltammetry and kinetic studies performed using ECL. The results showed that the enzymatic activity was significantly maintained as a result of the immobilization, with values of the apparent Michaelis–Menten constants estimated as 7.71 ± 0.62 and 8.41 ± 0.43 μM, for HRP and LOx, respectively. The proposed biosensors showed good stability and repeatability with an estimated limit of detection of 5.38 ± 0.05 and 0.50 ± 0.03 μM for hydrogen peroxide and lactic acid, respectively. The as-prepared enzymes encapsulated within the alginate hydrogels showed good stability up to 28 days from their preparation. The sensitivity and selectivity of the enzymes encapsulated within the alginate hydrogels were tested in real matrices (HRP, hydrogen peroxide, in contact lens solution; LOx, lactic acid in artificial sweat) showing the sensitivity of the ECL detection methods for the detection of hydrogen peroxide and lactic acid in real samples.

## 1 Introduction

Alginates are a class of biopolymers that have been used for various bioengineering and biomedical applications due to their biocompatibility, customizable and manageable properties, low toxicity, low cost, and mild gelation conditions.^1^ Alginates are derived from alginic acid sodium, which is a gelling and nontoxic anionic polysaccharide. Alginates, in combination to chitosan, have extensively been used in the tissue engineering and regenerative medicine fields to fabricate biodegradable porous scaffolds for bone tissues.^2^ These materials are cross-linked hydrophilic polymers that adsorb high quantity of water and still retaining the structure, despite their considerable swelling.^3−5^ The property of swelling is particularly useful in biomedical applications as this allows drugs to be transported inside the hydrogels and to be released selectively where it is required.^6,7^ The gelation of alginate is generally achieved by exchanging the sodium ions from the guluronic acid of the polymer chain with the divalent cations Ca^2+^.^8−11^ Hydrogel materials have also been used for various biotechnological applications like biosensing due to their biocompatibility, highly adaptable nature, and the significant property to swell in the presence of an aqueous environment. In particular, hydrogels based on natural polysaccharides have been employed for the development of enzymatic biosensors using different immobilization techniques.^12^ It is well known that hydrogels can be formulated in different ways including among others, microbeads. Fully biocompatible ionically cross-linked alginate hydrogels are very appealing as they offer the possibility to entrap active substances such as drugs and/or cells without affecting their biological activity. However, they present severe limitations such as leaching of the active materials from the inside of the highly hydrated porous structure and their burst release.^13^ Recently, Garcia-Rey et al. demonstrated the incorporation of enzymes such as horseradish peroxidase (HRP) and lactate and glucose oxidases for the enzymatic/colorimetric detection of lactic acid and glucose in artificial sweat.^14^ This procedure allowed detection of lactic acid and glucose in the millimolar range. Recently, two of us have shown that a model drug such as doxorubicin can successfully be entrapped within alginate microbeads in combination with nanostructured polyelectrolyte microcapsules by covering the drug-loaded CaCO_3_ microparticles with a polymeric shell.^15^ This approach was effective in preventing both leaching and burst release of the drug. In this work, we have adapted this approach to fabricate engineered alginate hydrogels for biosensing applications. Namely, we co-precipitated model enzymes, such as HRP and lactate oxidase (LOx), in CaCO_3_ microparticles and covered with a polymeric shell by the layer-by-layer (LbL) self-assembly technique. Enzyme immobilization in hydrogels may be based on encapsulation alone, but this is associated with enzyme leaching. To avoid this, covalent interactions can be established between the enzyme and the hydrogel matrix. However, covalent cross-linking could affect the enzyme activity, so that a mild approach is desirable. In this respect, in the present work, we propose the use of enzymatically active nanoengineered alginate^15−18^ but coupled with electrochemiluminescence (ECL) for the first time. The so modified CaCO_3_ microparticles were dispersed within alginate solution, followed by drop casting deposition on the glassy carbon electrode (GCE), gelation by dissolution of CaCO_3_ microparticles, and electrochemical detection using the electrogenerated chemiluminescence (ECL) produced in the presence of luminol as the luminophore.^19−21^ The chemiluminescence reaction between luminol and HRP is well established with HRP that has become the standard enzyme for chemiluminescent western blot detection, instead LOx is an enzyme belonging to the oxidases family that produces hydrogen peroxide as a result of the enzymatic reaction.^21^ Hydrogen peroxide is well known to be a coreactant for the ECL reaction with luminol; hence, the quantification of hydrogen peroxide allows the indirect detection of lactic acid, which is a reaction of relevance in many physiological processes.^22−24^ Noticeably, we used ECL to evaluate the enzymatic kinetic parameters using the Michaelis–Menten model as well as to assess the suitability of the as-prepared materials toward highly sensitive detection of glucose and lactic acid in real samples (contact lenses and artificial sweat solutions). We utilized the ECL as this analytical method is particularly attracting in electroanalysis due to its high sensitivity and selectivity, good reproducibility, and temporal and spatial control.^25−27^ ECL has also the significant advantage to discriminate against common electrochemical interferences.^28−31^ Finally, we coupled this analytical method to a hydrogel characterized by the presence of enzyme-loaded microvoids as a strategy of immobilization to guarantee stability and preservation of the enzyme functionality.^32^ Interestingly, the developed strategy could allow the accommodation of more recognition elements for the development of multifunctional biosensors.

## 2 Materials and Methods

### 2.1 Materials for Synthesis of Alginate Hydrogels

Alginic acid sodium salt from brown algae (Merck KGaA, Darmstadt, Germany), d-(+)-glucono-delta-lactone (GDL, Merck KGaA, Darmstadt, Germany), calcium chloride (CaCl_2_, Merck KGaA, Darmstadt, Germany), sodium carbonate (Na_2_CO_3_, Merck KGaA, Darmstadt, Germany), l-lactic acid (Merck KGaA, Darmstadt, Germany) at a concentration of 10 mM in deionized (DI) water and adjusted to a pH 7.4, luminol (97%, Merck KGaA, Darmstadt, Germany), LOx from *Aerococcus viridans* (LOx, 80 kDa, Merck KGaA, Darmstadt, Germany), HRP (Merck KGaA, Darmstadt, Germany), hydrogen peroxide (H_2_O_2_, extra pure, Fisher Scientific, Leicestershire, UK), phosphate-buffered saline (PBS, Merck KGaA, Darmstadt, Germany) were used. All stock solutions were prepared in the Milli-Q water purification system (18 mΩ cm).

### 2.2 Artificial Sweat

The artificial sweat solution was prepared by mixing 300 mM NaCl (99%, Merck KGaA, Darmstadt, Germany), 40 mM urea (≥98%, Merck KGaA, Darmstadt, Germany), 100 mM l-lactic acid, 100 mM d-(+) glucose (≥99.5%, Merck KGaA, Darmstadt, Germany), and 100 mL of distilled water and adjusted at pH 7.4. The solution was stored at 4 °C.^14^

### 2.3 Fabrication and Characterization of HRP and LOx-Loaded Alginate Hydrogels

**Scheme 1:**
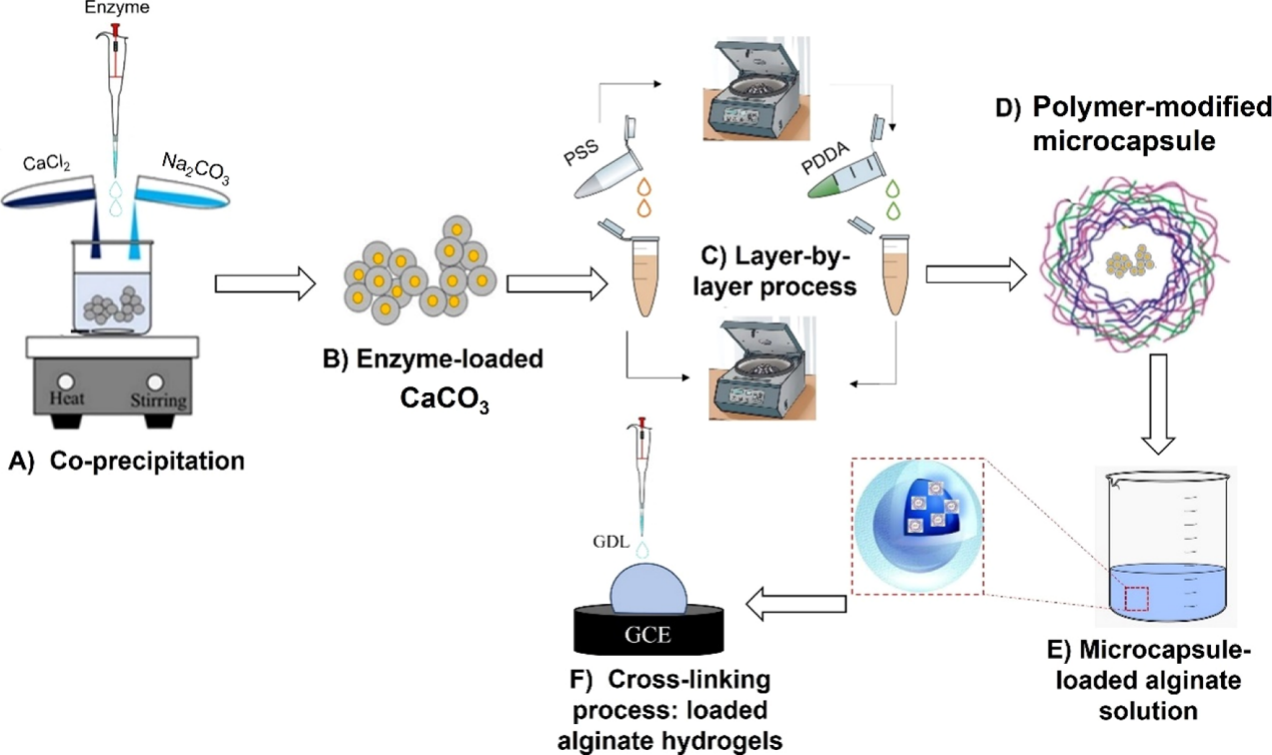
Schematic Representation of the Encapsulation of Enzyme into the Alginate Hydrogels; (A) Co-precipitation of Enzyme in CaCO_3_; (B) Enzyme-Loaded CaCO_3_; (C) LbL Deposition of Multi-layered Shell onto Enzyme-Loaded CaCO_3_; (D) Formation of Enzyme-Loaded Microcapsule; (E) Loading of Enzyme Microcapsules with Alginate Solution; and (F) Cross-Linking of Alginate-Modified Enzyme-Loaded Microcapsules

The alginate hydrogels were obtained by combining the following two procedures: (i) enzyme entrapment into microspheres and (ii) a LbL process.^33,34^ The entrapment of the enzymes was obtained by co-precipitation of the two salts, CaCl_2_ and Na_2_CO_3_, in the presence of the enzymes (HRP or LOx). Following previous studies,^33^ the microspheres fabrication consisted of mixing solutions of CaCl_2_ (0.33 M) and Na_2_CO_3_ (0.33 M). Precisely, 0.432 g of CaCl_2_ and 0.42 g of Na_2_CO_3_ were dissolved, respectively, in 12 mL of deionized water.^33,35^ A single procedure was performed mixing equal volumes of the two previous solutions (1 mL each) using a stirrer with a speed of 900 rpm for 20 s, obtaining CaCO_3_ particles in the vaterite form.^36^ After 20 s of stirring, the calcium carbonate templates were washed three times using deionized water. The supernatant was removed after using a centrifuge at 5000 rpm for 60 s leaving in this way just the microspheres. The entrapment of the enzymes by co-precipitation of the two salts in the presence of the enzymes followed the same steps of the CaCO_3_ core formation with the only difference consisting in the use of a volume of a solution of 1 mg/mL of the enzyme (HRP or LOx) in phosphate buffer, PBS, pH 7.4 and of 0.33 M Na_2_CO_3_ being added to an equal volume of 0.33 M CaCl_2_ under stirring (≈900 rpm), and after 20 s, stirring was stopped. By stirring the solution, it is possible to obtain the vaterite containing the enzyme. After the formation of the entrapped biomaterial, CaCO_3_ templates were covered by a LbL deposition of a shell made of two different polymers such as poly(sodium 4-styrenesulfonate), PSS, negatively charged, and poly(diallyldimethylammonium chloride), PDDA, positively charged. The preparation of the PSS polymer solution was performed by mixing 0.04 g of the polymer powder in 20 mL of deionized water allowing the preparation of a solution 0.2% wt, on the other side, the PDDA solution by mixing 250 μL of the polymer in 25 mL of deionized water. The LbL procedure was carried out by mixing the microspheres in 1 mL of the polymer solution starting with the PSS and alternating with the PDDA. For each layer, the use of a vortex for 15 min allowed us to strongly blend the compounds and to obtain the polyactive layer. It is advisable to repeat the procedure until reaching a number of six layers by an alternate use of PSS and PDDA according to Wang et al.^37^ Three washes with deionized water after each vortex were necessary using a centrifuge at 5000 rpm speed in order to remove the excess of the polymer before starting a new cycle of LbL deposition.^33^ For the preparation of alginate hydrogels, the following procedure was adopted: 1 g of the alginate sodium salt was dissolved into 50 mL of deionized water obtaining a 2% of alginic sodium salt solution.^15^ For an effective encapsulation of the enzyme-microsphere system, the mixing consists in 4 mL of alginic sodium salt 2% and 2 mL of template solution. The complete procedure is summarized in Scheme 1.

### 2.4 Confocal Microscopy

Confocal imaging was carried out to evaluate the distribution of microspheres into alginate hydrogels. The samples were prepared by loading into the CaCO_3_ template fluorescently labeled nanoparticles (FluoSpheres carboxylate-modified microspheres, *d* = 0.02 μm, red fluorescent, Thermo Fisher) instead of the enzyme molecules. The nanoparticles loaded templates were then covered by the LbL approach described above and then dispersed into the alginic acid solution. The alginate/microsphere dispersion was poured into a donut-shaped polydimethylsiloxane structure (internal and external diameters: 6 and 22 mm, respectively, height: 1 mm) and exposed d-glucono-1,5-lactone (GDL) (3% w/v in H_2_O) to dissolve the CaCO_3_ and thus releasing Ca^2+^ ions for the ionic gelation of alginic acid. Confocal imaging was acquired with a Leica TCS SP5 Tandem DMI6000 (Leica Microsystems CMS, Mannheim, Germany) inverted confocal laser scanning microscope coupled with Leica IRAPO 25×, 0.95 NA water immersion objective and Leica 63× PL APO1.4 NA oil immersion objective (Leica Microsystems, Mannheim, Germany).

### 2.5 Electrochemical Apparatus and Measurements

Electrochemical experiments were performed using a CH Instrument model 705E electrochemical potentiostat combined with a Hamamatsu H10721-20 photomultiplier tube (PMT). The electrochemical cell consisted of a 10 mL volume glass cell with a Teflon cover using a conventional three-electrode configuration consisting of a modified 3 mm working GCE and a platinum wire as the counter electrode purchased from IJ Cambria (UK). Potentials were quoted versus Ag/AgCl reference electrode. The ECL signal was detected by using a PMT, biased at 530 V using a high-voltage power supply circuit (model C10709), positioned against the electrochemical cell close to the working electrode. The electrochemical tests were carried out in 0.01 M PBS buffer solutions at pH 9 (for the HRP) and pH 7.4 (for the LOx). For both enzymes, the concentration of luminol used throughout the ECL experiments was 0.2 mM. The experiments were carried out by applying a potential in the range of 0–1 V and a scan rate of 0.05 V s^–1^. GCEs were polished by mechanical polishing of the surface using alumina slurry at different grades (100 and 50 μm) on polishing pads, rinsed, and sonicated with DI water to remove all the impurities. 3 μL of alginate hydrogels containing the encapsulated enzyme was drop cast onto the GCE surface. After a first dry of 2 h at room temperature, 3 μL of GDL was deposited on the hydrogel allowing the dissociation of the CaCO_3_ molecule with the consequent release of Ca^2+^ obtaining the core dissolution and the crosslink of the 3D network. Following the final dry at room temperature overnight, the modified GCEs were ready to be tested.^38^ For the stability tests, the enzymes incorporated within the alginate hydrogels and the free enzymes were stored for 28 days at 4 °C. The catalytic activity of the free and immobilized HRP and LOx was tested by recording the ECL curves every 2 days for a period of 28 days using the same aforementioned electrochemical conditions.

## 3 Results and Discussion

### 3.1 Confocal Microscopy

**Figure 1:**
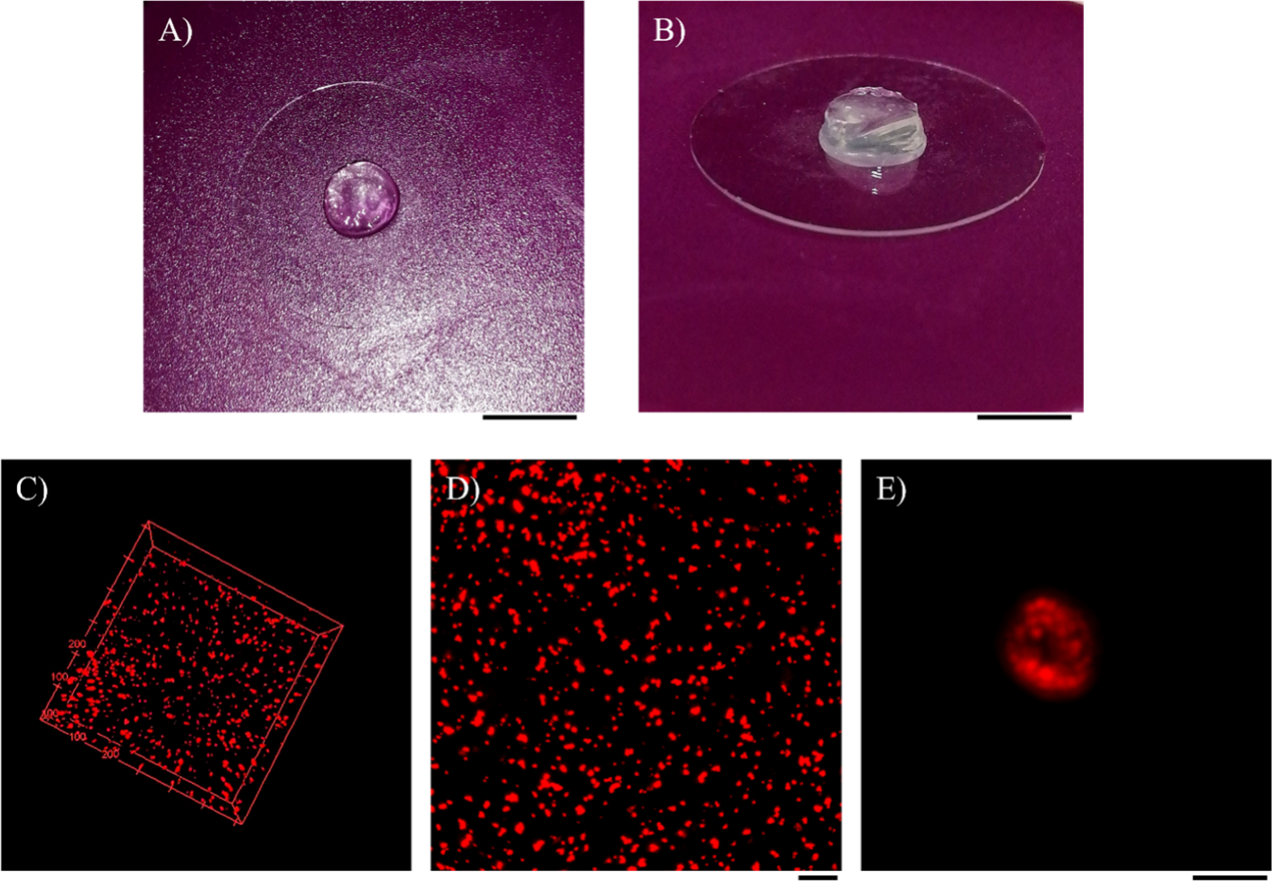
Photographs of alginate hydrogels (A,B); scale bar: 5 mm. (C–E) Confocal images: (C) rendering 3D of microcapsule dispersion into alginate hydrogel (*z* = 500 μm). (D) Max intensity projection of 500 μm *z*-stack of microcapsules embedded into alginate hydrogel; scale bar: 50 μm; and (E) max intensity projection of a single microsphere embedded into alginate hydrogel; scale bar: 4 μm.

The distribution of the microcapsule-loaded hydrogels was observed by confocal images. The alginate hydrogel appeared transparent (Figure 1A,B). The 3D reconstruction and the max projection of 500 μm *z*-stack of the hydrogel suggest that microcapsules appeared highly uniform and homogeneously distributed within the hydrogel (Figure 1C,D). Figure 1E shows a 3D reconstruction of the fluorescent nanospheres suspended through the matrix of the hydrogel sample. Confocal *z*-stacks were also converted to the movie that depicts the 3D distribution and resolution of the microcapsules (see Supporting Information S1, file hydrogel.avi).

### 3.2 Electrochemical Characterization

**Figure 2:**
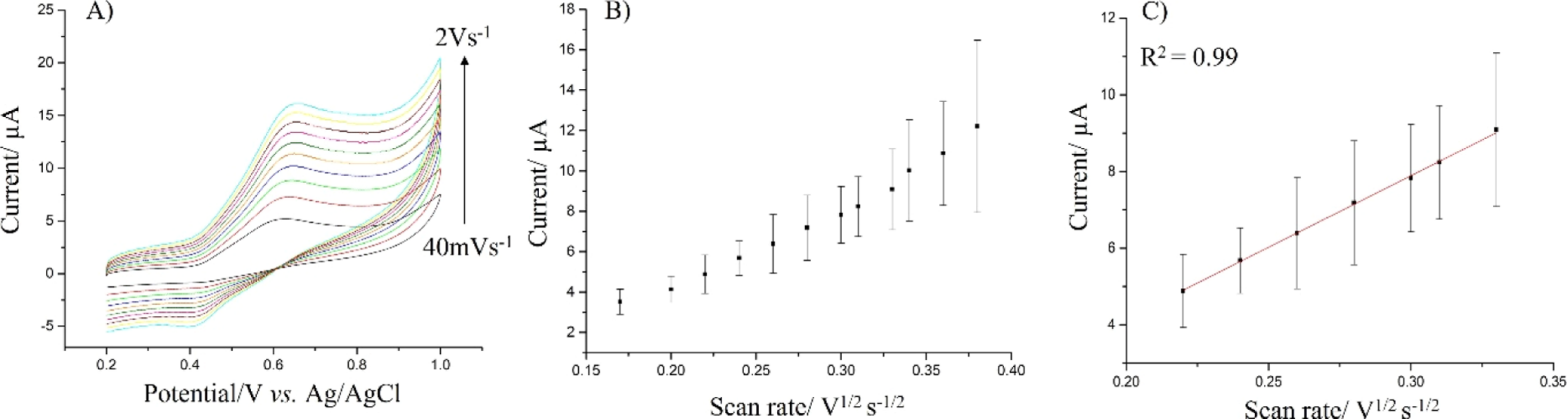
CVs of luminol at alginate hydrogel-modified GCE at different scan rates from 0.04 to 2 V s^–1^ (A); plot of the anodic peak currents vs the square root of the scan rate (B); and other conditions: supporting electrolyte 0.01 M PBS (pH 9) containing 0.2 mM luminol. The linear range is taken from 0.17 to 0.33 V^1/2^ s^–1/2^ of the square root of the scan rate (corresponding to scan rates in the range 0.03–0.1 V s^–1^) (C). Error bars represent triplicate data repetitions.

At first, cyclic voltammetry (CV) was utilized to ascertain the diffusion of both substrate (H_2_O_2_) and luminophore (luminol) within the alginate hydrogels. For instance, the CVs with a fixed concentration of luminol (2.5 mM) were recorded. Figure 2 shows the CVs obtained at different scan rates (from 0.04 to 2 V s^–1^) in 2.5 mM luminol and 0.01 M PBS-supporting electrolyte (pH = 9). We selected pH 9 as the ECL of luminol-based systems requires very basic pH values (typically pH values >9), and this is well established for luminol-based systems.^39^ The CVs show the typical oxidation peak of luminol at ca. 0.65 V and reduction at 0.4 V, with the oxidation peak currents scaling linearly with the square root of the scan rate according to the Randles–Sevcik equation, as expected for a diffusion-controlled process^40^
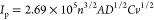
1where *I*_p_ is the oxidation peak current (*A*); *n* is the number of electrons transferred during the reactions; *F* is the Faraday constant (96,485 C mol^–1^); *A* is the area of the electrode (cm^2^); *C* is the concentration of the luminophore (mol cm^–3^); and *v* is the scan rate (V s^–1^).^40^ This finding is not unexpected as luminol diffuses easily within the alginate hydrogels as a result of its porous structure with a diffusion coefficient estimated from the Randles–Sevcik equation as *D* = (5.63 ± 0.8 × 10^–6^) cm^2^ s^–1^.

**Figure 3:**
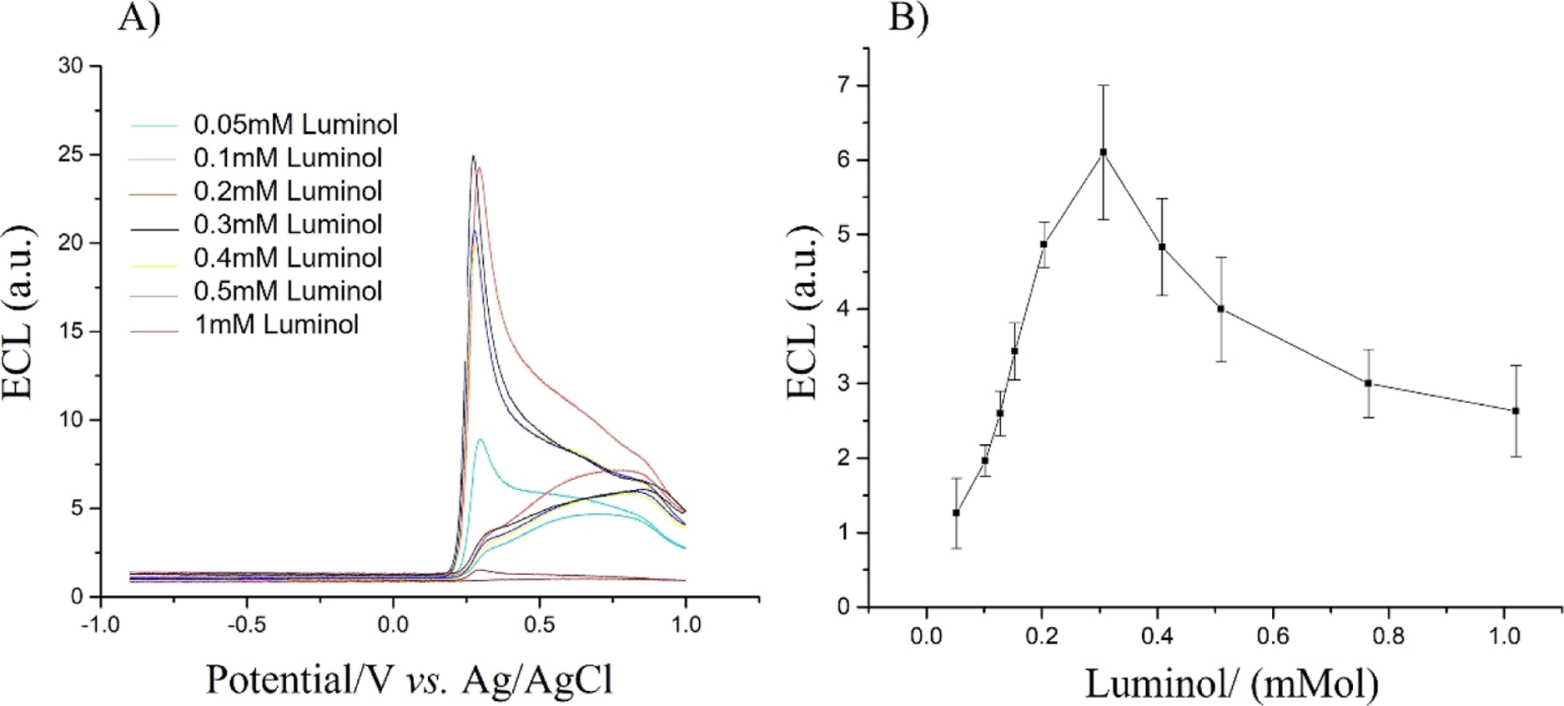
ECL curves vs concentration of luminol from 0.05 to 1 mM recorded in 0.01 M PBS (pH 12) containing 22 μM of H_2_O_2_; scan rate 0.05 V s^–1^ (A). Plot of ECL signal vs concentration of luminol (B). Error bars represent triplicate data repetitions.

Before proceeding with the ECL characterization of the enzymes encapsulated within the alginate hydrogels, an experiment was carried out to find the concentration of luminol that provides a higher ECL signal. Figure 3 shows the ECL curve obtained at different concentrations of luminol and maintaining a constant concentration of coreactant (H_2_O_2_, 22 μM) with a value of pH = 12 as the p*K*_a_ of hydrogen peroxide is 11.75 which corresponds to high values of ECL emission of luminol in alkaline media.^41^ A concentration of 10 μL (22 μM) was selected as at this concentration, the ECL signal provided a reasonable readable signal (figure not shown). The plot of the ECL versus the concentration of luminol shows that the highest ECL signal is reached using a 0.3 mM concentration of luminol, whereas for higher concentrations of luminol, the ECL signal decreases. To note that at concentrations of luminol higher than 0.3 mM, the standard deviation increases considerably. For this reason, a 0.2 mM concentration of luminol is selected as the optimum concentration for all following experiments. This selected value is in agreement with the work of Chen et al.^42^

**Scheme 2:**
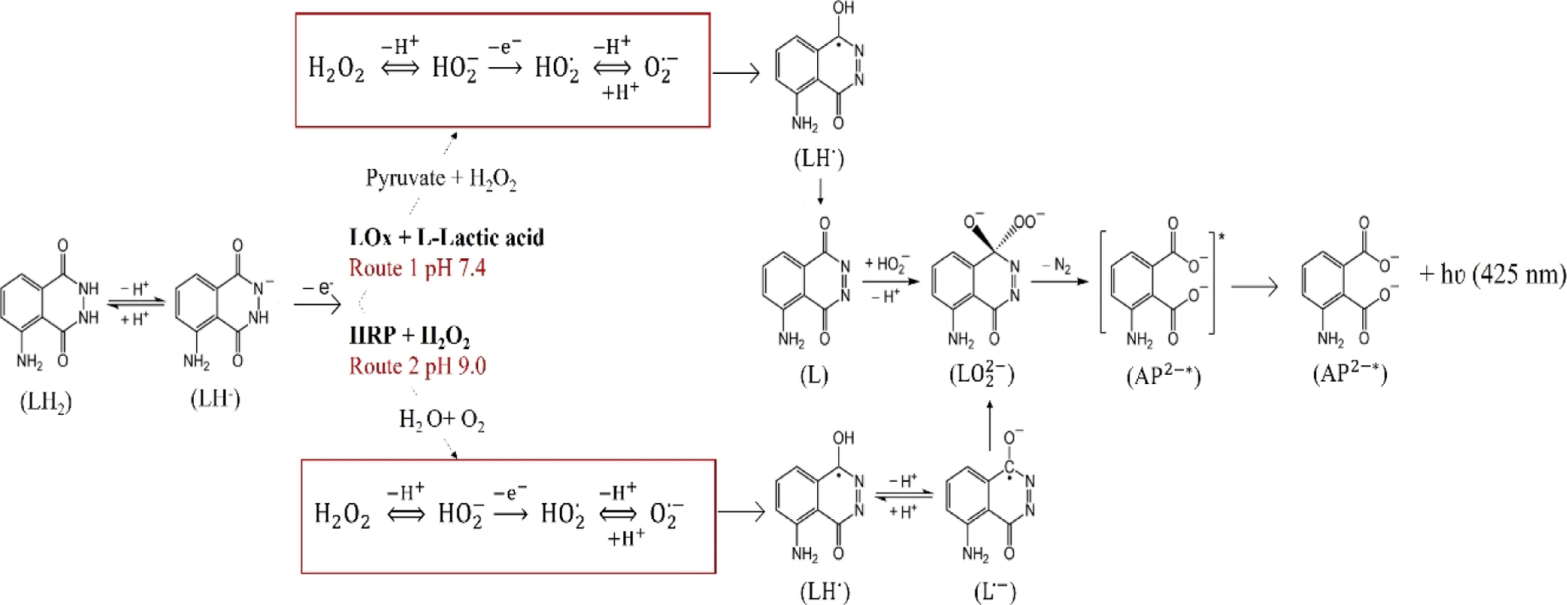
Schematic Mechanism of ECL Generation by Luminol/H_2_O_2_ Coupled with the Enzymatic Reaction of HRP and LOx at the Corresponding pH

The emission of light occurs according to a well-established mechanism summarized in Scheme 2. The chemiluminescent reaction of luminol needs a basic pH to occur as in these conditions, the base removes the nitrogen protons leaving a negative charge that forms an enolate after moving onto the carbonyl oxygen. Cleavage of the hydrogen peroxide performed by HRP leads to the formation of a highly reactive oxygen species that performs a cyclic addition to the carbonyl carbons, causing the formation of an excited intermediate 3-APA* which decays with emission of radiation at 425 nm, and molecular nitrogen as a leaving group. In the case of LOx, when a suitable potential of 0–1 V is applied to the electrode, both luminol and H_2_O_2_ undergo oxidation forming luminol radicals and superoxide radical anions. At pH > 9, the luminol radical is deprotonated forming a luminol radical anion that reacting with the superoxide radical anion leads to the formation of LO_2_^2–^. Decomposition of LO_2_^2–^ yields to the formation of the 3-APA* with emission of light at 425 nm.^43^

**Figure 4:**
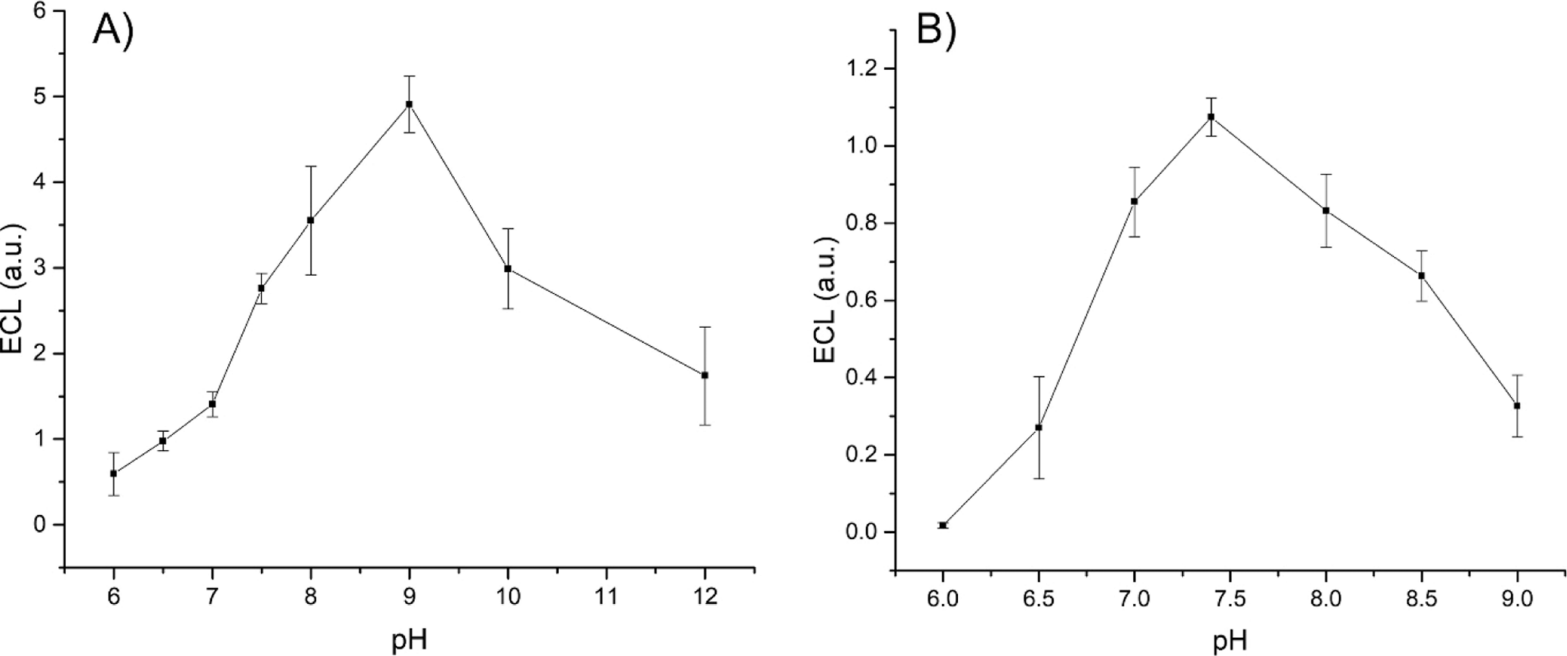
Effect of pH on the ECL response of HRP/H_2_O_2_/luminol (A) and LOx/l-lactic acid/luminol (B) recorded in 0.01 M PBS-supporting electrolyte containing 0.2 mM luminol. The concentration of the substrate was 22 μM hydrogen peroxide (A) and 30 μM l-lactic acid (B); scan rate 0.05 V s^–1^. Error bars represent triplicate data repetitions.

Typically, the enzymatic activity is greatly affected by the pH, and as the ECL of luminol requires alkaline values of pH, in a following experiment, a pH study was carried out in order to find the value of pH at which the ECL emission is at the maximum. Figure 4 illustrates the ECL response obtained in 0.01 M PBS in the range of pH 6–12. As expected, the ECL response is very weak at pH 6 and increases gradually reaching a maximum for values of pH as 9 and 7.5 for HRP and LOx, respectively. For values of pH higher than 9 and 7.5, the ECL response decreases noticeably as an indication that at very alkaline values of pH, the enzymatic activity is hindered. These values agree well with the data available in the literature, which show that the maximum enzymatic activity for HRP and LOx occurs at 9 and 7.4 pH, respectively.^44,45^ Based on that, the following experiments with HRP and LOx were carried out at pH 9 and 7.4, respectively.

**Figure 5:**
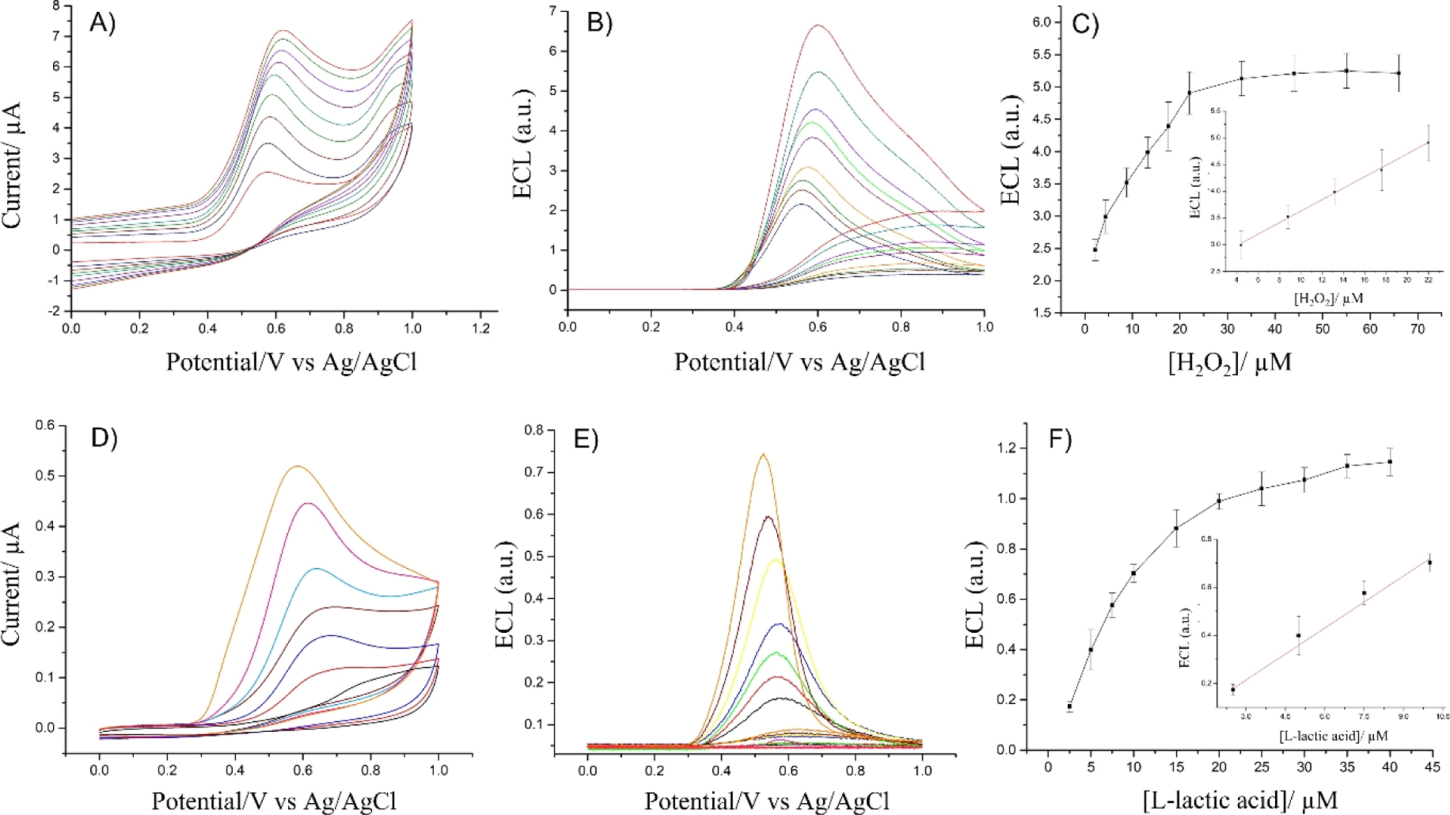
CVs and ECL response of the HRP–luminol/H_2_O_2_ system (A,B) and of LOx/luminol/lactic acid (D,E) vs concentrations of hydrogen peroxide and lactic acid recorded in 0.01 M PBS (pH 9.0 for the HRP/luminol/H_2_O_2_ system and pH 7.4 for LOx/luminol/lactic acid); scan rate 0.05 V s^–1^. Plot of ECL vs concentration of hydrogen peroxide (C) and l-lactic acid (F). Error bars represent triplicate data repetitions.

To establish whether the HRP and LOx incorporated within the alginate hydrogels are suitable for the voltammetric and ECL detection of hydrogen peroxide and lactic acid, several CVs and ECL curves were recorded with the addition of different concentrations of H_2_O_2_ and lactic acid. The experiments were performed in 0.01 M PBS at pH 9 for HRP and pH 7.4 for LOx. By increasing the concentration of the substrate in solution, the amount of oxygen and hydrogen peroxide produced as a result of the corresponding enzymatic reaction increases, generating a higher ECL signal due to the reaction with luminol. Figure 5 shows that in the case of HRP, the ECL signal increases linearly with the concentration of hydrogen peroxide up to 20 μM, then the ECL emission plateaus, whereas for LOx, the ECL signal increases up to a concentration of 20 μM of lactic acid added, plateauing for concentrations higher than 25 μM. The limit of detection (LOD) is calculated following the 3σ_b_/slope criteria considering σ_b_ as the standard deviation of the intercept. The estimated values of LOD are 0.38 ± 0.5 μM for HRP and 0.35 ± 0.4 μM for LOx. These results show that the enzymes encapsulated within the alginate hydrogels are promising candidates for highly sensitive detection of hydrogen peroxide and lactic acid. The trend of the ECL signal versus the substrate concentration suggests the possibility to evaluate the enzymatic activity by fitting the ECL data with the Michaelis–Menten equation which will be investigated in the next section.

**Figure 6:**
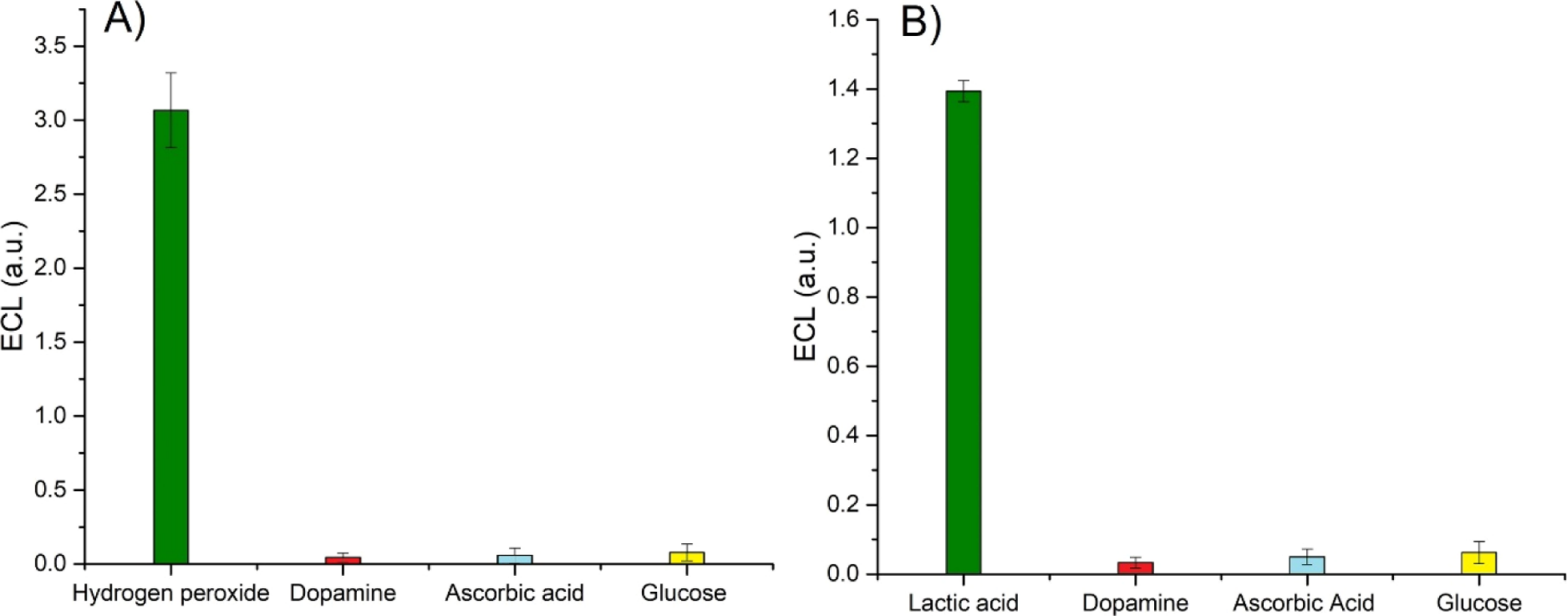
Dependence of the ECL signal for the HRP/luminol/GCE (A) and LOx/luminol/GCE (B) recorded in 0.01 M PBS after addition of 0.1 mM dopamine, 0.1 mM ascorbic acid, and 0.1 mM glucose as interference species. The concentrations of the substrates were 22 and 30 μM of hydrogen peroxide and lactic acid, respectively. Error bars represent triplicate data repetitions.

An important aspect to determine is whether the electrochemical detection can be affected by the presence of potential interferences. For this reason, the ECL emission was tested in the presence of common interferences such as dopamine, ascorbic acid, and glucose whose concentrations were well in excess and set to 0.1 mM compared to the concentration of the substrate. The results are reported in Figure 6 and show that the ECL emission is not significantly affected by the presence of the interference species, evidencing the suitability for applications in real samples.

### 3.3 Stability of Enzymes Encapsulated within Alginate Hydrogels

**Figure 7:**
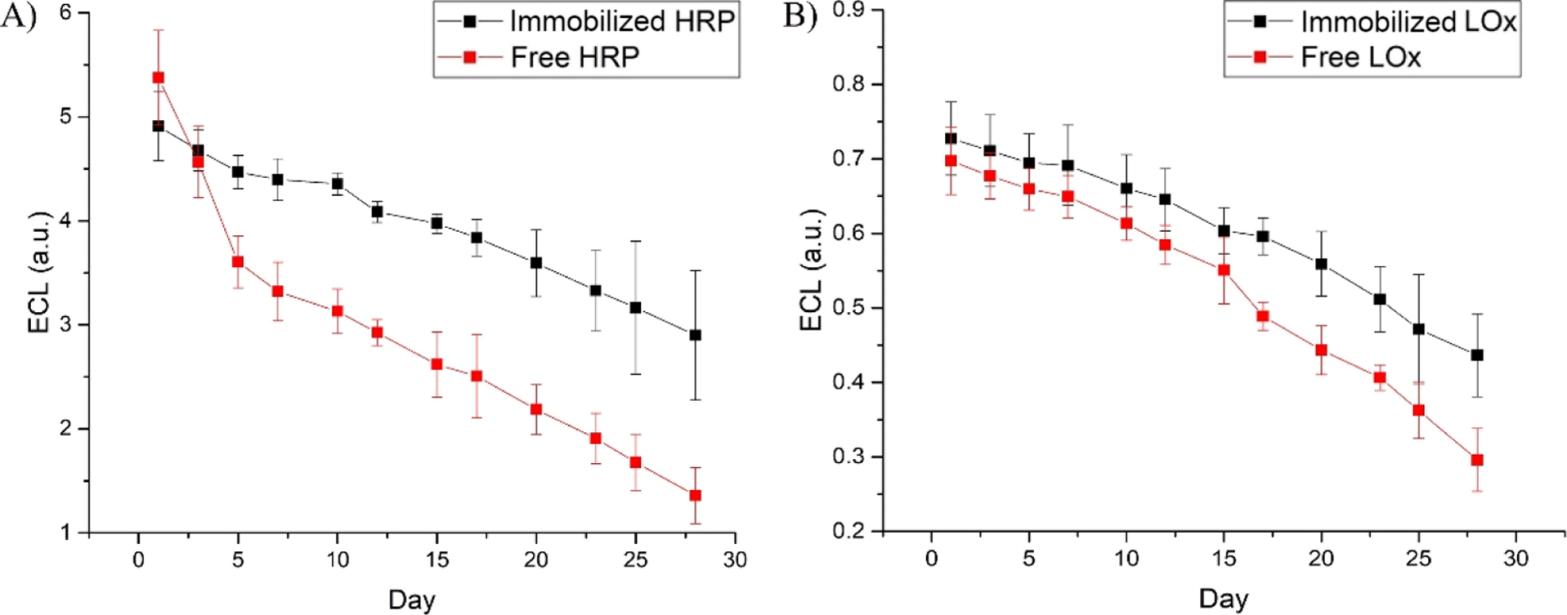
ECL curves vs time for HRP (A) and LOx (B) immobilized (black line) within the alginate hydrogels and the corresponding free enzyme in solution (red line). The ECL curves were recorded in 0.01 M PBS pH 9.0 for the HRP–H_2_O_2_ system and pH 7.4 for the LOx–l-lactic acid system; scan rate 0.05 V s^–1^. The concentration of substrates (H_2_O_2_ and l-lactic acid) were 22 and 30 μM, respectively. Error bars represent triplicate data repetitions.

The stability of the enzymes encapsulated within the alginate hydrogels was assessed by studying the ECL signal over the time. The results reported in Figure 7 show that for both HRP and LOx, the catalytic activity is maintained with a decrease in the ECL signal of ca. 3% every 2 days and an overall loss of ca. 30% after 28 days, whereas the corresponding ECL signal of the free enzymes decreases more dramatically, for example, 40% in the first four days with a loss that reached 80% at the end of 28 days. Noticeably, the catalytic activity is always higher in the encapsulate enzymes as an indication that the immobilization within the alginate hydrogels improves the enzyme stability. Despite the loss of enzymatic activity over the time which is widely expected, the ECL signals of the immobilized enzymes are still easily measurable after 28 days as a proof of the effectiveness of the immobilization procedure and of the enhanced sensitivity of the ECL method.

### 3.4 Evaluation of the Kinetic Parameters through ECL Analysis

**Figure 8:**
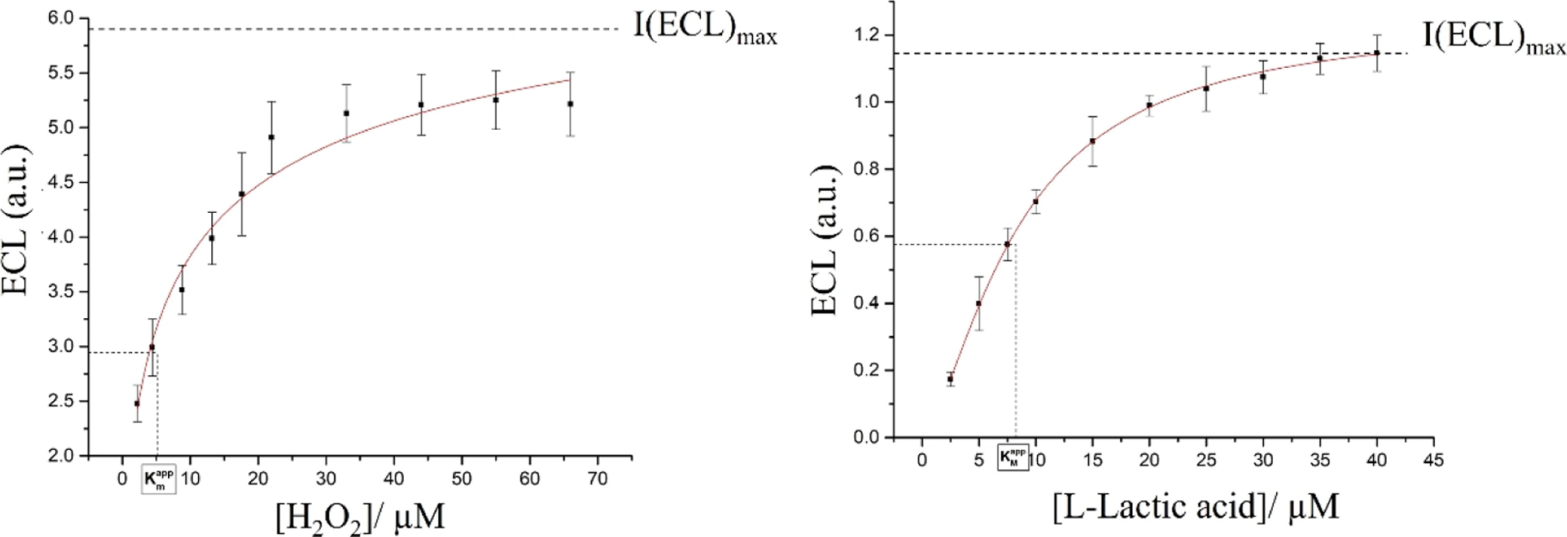
ECL vs substrate concentration (dots) and corresponding Michaelis–Menten fit (line) showing the  and the *I*_max(ECL)_ value at which the enzymes reach the saturation point. Error bars represent triplicate data repetitions.

It is well established that the best method to assess the enzymes kinetics is represented by the Michaelis–Menten method, which is widely used to predict the enzyme activity. Typically, the Michaelis–Menten plots report the measured reaction velocities versus the substrate concentration. In our case, the enzymatic activity is related to the enzyme as the catalyst for the oxidation reactions related to HRP and LOx. As a result, the number of electrons released is proportional to the substrate molecules; hence, not only the oxidation peak current (CV) but also the ECL emission are measured for additional substrate concentrations. In this case, rather than plotting the initial rates versus the substrate concentration, it is possible to plot the corresponding ECL emission versus the substrate concentration.^46^ To note that the emission of light as a result of the enzymatic reaction with luminol is read by the potentiostat as a signal of current, hence the following equation applies^47^
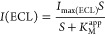
2where  is the maximum obtainable ECL intensity, *S* is the concentration of the substrate, and  is the apparent Michaelis–Menten constant. For both the enzymes, the  values were calculated, considering the maximum ECL intensity that each enzyme can achieve and the substrate concentration at which the ECL intensity corresponds to the 50% of the *I*_max(ECL)_.^48^ The results, as illustrated in Figure 8, show a good fit between the ECL emission versus substrate concentration using the Michaelis–Menten model. This suggests the possibility to estimate the  values to evaluate the enzymatic activity of HRP and LOx immobilized within the alginate hydrogels. The calculated  values were found to be 7.71 ± 0.62 μM for HRP and 8.41 ± 0.43 μM for LOx. These values show that the enzymatic activity of HRP and LOx immobilized within the alginate hydrogel is retained and much higher than other values found in the literature from other methods of enzyme immobilization (see Supporting Information S2). We underline that small  values indicate a high affinity of the enzyme and therefore a strong binding with the corresponding substrate. Therefore, the proposed biosensor provides a biocompatible environment for both enzymes to retain their structure and activity. This fact suggests that both HRP and LOx encapsulated within the alginate hydrogels maintain a high degree of freedom, with values of  not much dissimilar from the values of *K*_M_ of both free enzymes in solution.^49,50^

### 3.5 ECL Detection of Hydrogen Peroxide and Lactic Acid in Real Samples

**Figure 9:**
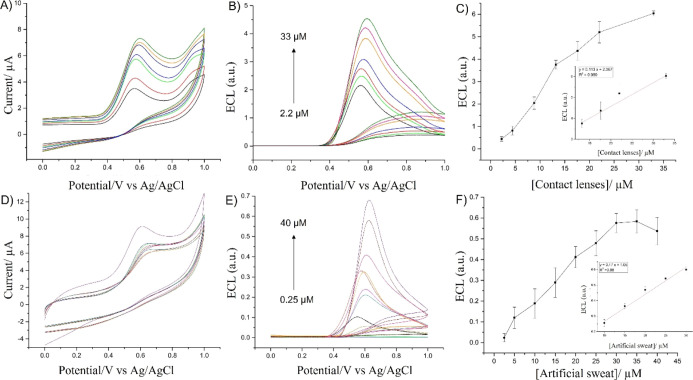
CVs (A) and ECL curves (B) of HRP/H_2_O_2_/luminol/GCE recorded at increased aliquots of contact lens liquid; supporting electrolyte: 0.01 M PBS pH 9.0, scan rate 0.05 V s^–1^. CVs (D) and ECL curves (E) of LOx/luminol/GCE recorded at the increased concentrations of artificial sweat, supporting electrolyte: 0.01 M PBS pH 7.4, scan rate 0.05 V s^–1^. Plots of the ECL signal vs contact lens solution (C) and artificial sweat (F). Error bars represent triplicate data repetitions.

Finally, to test the suitability of the as-prepared modified electrodes in real samples, we tested the HRP/luminol/GCE for the detection of hydrogen peroxide in commercial contact lens liquid, whereas the LOx/luminol/GCE was utilized for the detection of lactic acid in the artificial sweat sample. Both the real samples were diluted in 0.01 M PBS obtaining a stock solution of 20 mM concentration. The results shown in Figure 9 show that, as expected, the anodic peak currents and the related ECL signals increase with the amount of contact lens solution and artificial sweat added to the electrolytic solution. The ECL signals related to HRP (Figure 9C) and LOx (Figure 9F) show a linear dependence over the range 13–33 μM in contact lens liquid and 10–30 μM in artificial sweat. The calculated detection limits, LOD, are 0.57 ± 0.8 μM (HRP) and 1.9 ± 0.3 μM (LOx), showing that the as-prepared materials can detect very low concentrations of hydrogen peroxide and l-lactic acid, respectively.

## 4 Conclusions

We have demonstrated that HRP and LOx can efficiently be incorporated within alginate hydrogels. The procedure involved the incorporation of the enzymes withing Na_2_CO_3_ microspheres followed by incorporation within the alginate hydrogels. For the first time, we have utilized the ECL for the estimation of the enzymatic activity of HRP and LOx within the alginate hydrogels. The results showed that the ECL data fitted the Michaelis–Menten model with the values of the apparent Michaelis–Menten constant  calculated as 7.71 ± 0.62 and 8.41 ± 0.43 μM for HRP and LOx, respectively. These results showed that the enzymatic activity is well retained and with the values of  obtained to be among the best when compared with other methods of immobilization of the same enzymes. Finally, HRP and LOx incorporated within the alginate hydrogels were tested for the detection of hydrogen peroxide and l-lactic acid using ECL detection with luminol as the luminophore. The results showed the high sensitivity of the ECL reaching detection limits as low as 0.57 ± 0.8 and 1.9 ± 0.3 μM for hydrogen peroxide and l-lactic acid, respectively. These results point out that the alginate hydrogels are a good and stable platform for the immobilization of enzymes by maintaining a higher enzymatic activity compared to other immobilization methods. We believe this platform could be useful in the biocatalysis of industrial processes.

## Supporting Information Available

3D resolution of microcapsules (AVI)
Comparison of Michaelis–Menten constants, linearity range, and LOD values for HRP and LOx deposited on electrodes with different immobilization strategies (PDF)

## Supplementary Material

ac2c03389_si_001.avi
