# Supplementary material for: Enzymes Encapsulated within Alginate Hydrogels: Bioelectrocatalysis and Electrochemiluminescence Applications
Source: Anal Chem. 2022 Nov 8;94(46):16122–31. doi: 10.1021/acs.analchem.2c03389 (PMC9685591; doi:10.1021/acs.analchem.2c03389)
Supplement: Supplementary file 2 — ac2c03389_si_002.pdf [file ac2c03389_si_002.pdf]

Supporting Information

Enzymes-encapsulated within Alginate Hydrogels: Bioelectrocatalysis and Electrochemiluminescence Applications

Lucia Simona Ferraraccio,[a,b] Donatella Di Lisa, [c] Laura Pastorino,[c] and Paolo Bertoncello,[a,b] *

[a] Department of Chemical Engineering, Faculty of Science and Engineering, Swansea University, Bay Campus, Crymlyn Burrows, Swansea SA1 8EN, United Kingdom

[b] Centre for NanoHealth, Swansea University, Singleton Campus, Swansea SA2 8PP, United Kingdom

[c] University of Genova, Department of Informatics, Bioengineering, Robotics and System Engineering, Via Opera Pia 13, 16145 Genova, Italy

*corresponding author (p.bertoncello@swansea.ac.uk)

S1

Video related to the microscopy of the enzymes encapsulated into Alginate Hydrogels: hydrogel.avi

Table S1

Comparative table of Michaelis-Menten constants, linearity range and limits of detection values for HRP and LOx deposited on electrodes with different immobilisation strategies.

**Immobilized enzyme**	**Sensitivity ($\mu A\ mM^{-1}\ cm^{-2}$)**	**Technique**	**Linear range**	**LOD (µM)**	$\boldsymbol{K_m^{app}}$ **(mM)**	**Ref.**
HRP	Nc	ECL	0.1 nM–10 mM	--	0.5	[1]
HRP	Nc	CV	1.4 µM–2.8 mM	0.58	2.3	[2]
HRP	nc	CV	0.48 – 50 µM	0.21	3.69 ± 0.71	[3]
HRP	1.513	ECL	4 – 22 µM	0.38 ± 0.5	$(7.71 \pm 0.62) \times 10^{-3}$	This work
LOx	(4.5 ± 6)	CV	0 – 5 mM	3	2.3 ± 0.3	[4]
LOx	(18.9 ± 4.8)	CA	0 – 1 mM	193 ± 41	0.183 ± 0.66	[5]
LOx	(36.8×10^{3})	CV	25 – 1500 µM	11	1.6	[6]
LOx	1020	CV	--	0.5	0.88 ± 0.07	[7]
LOx	1.018	ECL	2.5 – 10 µM	0.35 ± 0.4	$(8.41 \pm 0.43) \times 10^{-3}$	This work

ECL= Electrochemiluminescence; CV=cyclic voltammetry; CA= chronoamperometry; nc= not calculated

References

1. Zhang, Q.; Xu, G.; Gong, L.; Dai, H.; Zhang, S.; Li, Y.; Lin, Y., An enzyme-assisted electrochemiluminescent biosensor developed on order mesoporous carbons substrate for ultrasensitive glyphosate sensing. *Electrochimica Acta* **2015,** *186*, 624-630.
2. Yi, X.; Huang-Xian, J.; Hong-Yuan, C., Direct Electrochemistry of Horseradish Peroxidase Immobilized on a Colloid/Cysteamine-Modified Gold Electrode. *Analytical Biochemistry* **2000,** *278* (1), 22-28.
3. Liu, S.-Q.; Ju, H.-X., Renewable reagentless hydrogen peroxide sensor based on direct electron transfer of horseradish peroxidase immobilized on colloidal gold-modified electrode. *Analytical Biochemistry* **2002,** *307* (1), 110-116.
4. Hickey, D. P.; Reid, R. C.; Milton, R. D.; Minteer, S. D., A self-powered amperometric lactate biosensor based on lactate oxidase immobilized in dimethylferrocene-modified LPEI. *Biosensors and Bioelectronics* **2016,** *77*, 26-31.
5. Taurino, I.; Reiss, R.; Richter, M.; Fairhead, M.; Thöny-Meyer, L.; De Micheli, G.; Carrara, S., Comparative study of three lactate oxidases from Aerococcus viridans for biosensing applications. *Electrochimica Acta* **2013,** *93*, 72-79.
6. Lamas-Ardisana, P. J.; Loaiza, O. A.; Añorga, L.; Jubete, E.; Borghei, M.; Ruiz, V.; Ochoteco, E.; Cabañero, G.; Grande, H. J., Disposable amperometric biosensor based on lactate oxidase immobilised on platinum nanoparticle-decorated carbon nanofiber and poly (diallyldimethylammonium chloride) films. *Biosensors and Bioelectronics* **2014,** *56*, 345-351.
7. Iwuoha, E. I.; Rock, A.; Smyth, M. R., Amperometric l-lactate biosensors: 1. Lactic acid sensing electrode containing lactate oxidase in a composite poly-l-lysine matrix. *Electroanalysis: An International Journal Devoted to Fundamental and Practical Aspects of Electroanalysis* **1999,** *11* (5), 367-373.
